# Supplementary material for: Digital Serious Games for Cancer Education and Behavioural Change: A Scoping Review of Evidence Across Patients, Professionals, and the Public
Source: Cancers (Basel). 2025 Oct 18;17(20):3368. doi: 10.3390/cancers17203368 (PMC12563440; doi:10.3390/cancers17203368)
Supplement: Supplementary file 1 [file cancers-17-03368-s001.zip › Supplementary Material 2 Search Strategy.pdf]

Supplementary Materials 1-Search Strategy

#	Query
**WEB OF SCIENCE**	
1	(serious game* OR gamification OR computer game* OR digital game* OR health game* OR game*).mp.
2	(cancer* OR neoplasm* OR oncology OR tumor OR tumour OR malignancy OR carcinoma).mp.
3	(awareness OR knowledge OR education OR learning OR understanding OR literacy OR prevention OR detection OR screening OR treatment OR intervention).mp
4	1 AND 2 AND 3
5	limit 4 to English language

#	Query
**MEDLINE**	
1	(serious game* OR gamification OR computer game* OR digital game* OR health game* OR game*).mp.
2	Exp Gamification
3	1 OR 2
4	(cancer* OR neoplasm* OR oncology OR tumor OR tumour OR malignancy OR carcinoma).mp.
5	Exp (Neoplasms OR Carcinoma)
6	4 OR 5
7	(awareness OR knowledge OR education OR learning OR understanding OR literacy OR prevention OR detection OR screening OR treatment OR intervention).mp
8	Exp (Awareness OR Knowledge OR Education OR Learning OR Comprehension OR Literacy)
9	7 OR 8
10	3 AND 6 AND 9
11	limit 10 to English language

#	Query
**CINAHL**	

1	(serious game* OR gamification OR computer game* OR digital game* OR health game* OR game*).mp.
2	Exp (Gamification OR Games)
3	1 OR 2
4	(cancer* OR neoplasm* OR oncology OR tumor OR tumour OR malignancy OR carcinoma).mp.
5	Exp (Neoplasms OR Oncology OR Carcinoma)
6	4 OR 5
7	(awareness OR knowledge OR education OR learning OR understanding OR literacy OR prevention OR detection OR screening OR treatment OR intervention).mp
8	Exp (Knowledge OR Education OR Learning OR Literacy)
9	7 OR 8
10	3 AND 6 AND 9
11	limit 10 to English language

#	**Query**
**PSYCINFO**	
1	(serious game* OR gamification OR computer game* OR digital game* OR health game* OR game*).mp.
2	Exp Gamification
3	1 OR 2
4	(cancer* OR neoplasm* OR oncology OR tumor OR tumour OR malignancy OR carcinoma).mp.
5	Exp (Neoplasms OR Carcinoma)
6	4 OR 5
7	(awareness OR knowledge OR education OR learning OR understanding OR literacy OR prevention OR detection OR screening OR treatment OR intervention).mp
8	Exp (Awareness OR Knowledge OR Education OR Learning OR Comprehension OR Literacy)
9	7 OR 8
10	3 AND 6 AND 9
11	limit 10 to English language
