# Supplementary material for: Digital Serious Games for Cancer Education and Behavioural Change: A Scoping Review of Evidence Across Patients, Professionals, and the Public
Source: Cancers (Basel). 2025 Oct 18;17(20):3368. doi: 10.3390/cancers17203368 (PMC12563440; doi:10.3390/cancers17203368)
Supplement: Supplementary file 1 [file cancers-17-03368-s001.zip › Supplementary Material 3 Included Study Characteristics.pdf]

Characteristics of Included Studies

Study design	Author (year), country	Study objectives	Study methods	Population characteristics	Game type	Key findings	COM-B model components
Qualitative (n=3)	Horsham et al. (2021) Australia	To develop a virtual reality (VR) game containing preventive skin cancer messaging. To assess the safety and satisfaction of the design based on end user feedback.	Focus group and interview	18 participants aged 18 to 74 years old from the public in total. 11/18 participants had never experienced VR game before. 2/18 participants had been diagnosed with skin cancer.	Virtual Reality game	Qualitative feedback indicated high levels of satisfaction, with all participants reporting the VR game as engaging.<br><br>A total of 11% (2/8) of participants reported a side effect of feeling nauseous during the experience.<br><br>The end user feedback identified game improvements, suggesting an extended multistage	Opportunity Motivation

						experience with visual transitions to other environments and interactions involving cancer causation.<br><br>The implementation of the VR game identified challenges in sharing VR equipment and hygiene issues.	
	Oliveira et al. (2024)<br>Brazil	To improve awareness and educate players about managing thyroid cancer through interactive gameplay.	Qualitative assessment form	3 doctors who were thyroid health specialists	Digital Tamagotchi-style serious game	The game has engaging visuals, accurate portrayal of thyroid diseases, and educational value.	Motivation
	Brown-Johnson et al. (2015)<br>United States of America	To test the feasibility and usability of mHealth TLC, an interactive, immersive 3-dimensional iPad health game that	Semi-structured interview	8 health professionals, including nurses, doctors, and researchers	Interactive health game	All participants confirmed mHealth TLC to be: (1) believable, (2) clinic-appropriate,	Motivation

		coaches lung cancer patients toward assertive communication strategies during first-person virtual clinics visits.				and (3) helpful in support of informed healthcare consumers.<br><br>Concerns were expressed about emotionally charged content.<br><br>Although the dialog and interactions addressed emotionally charged issues, players were able to engage, learn, and benefit from role-play in a virtual world. Health games have the potential to improve patient–clinician communication, and mHealth TLC specifically may	

						decrease lung cancer stigma, and promote optimal self-management.	
Quantitative (n=18)	Mettarikanon et al. (2023) Thailand	To evaluate and compare cognitive performances pertaining to fundamental knowledge of cutaneous malignancies and their perspectives between the participants in the game group and those in the pamphlet group.	Quasi-experimental research design. Pre- and post-test.	A total of 94 participants from the public. All of them were undergraduate non-medical students. Their mean age was 19.8 years old.	Online web-based game	Online game was a promising method for enhancing recognition of common cutaneous malignancies. With repetitive practice, the participants significantly gained favorable skills with a high level of satisfaction.<br><br>The post-intervention survey in the game group demonstrated a high degree of satisfaction, particularly in self-reported joyfulness of playing the game	Capability Opportunity

						compared to that of reading the pamphlet.	
	Carcioppolo et al. (2022) United States of America	To develop and evaluate an online, game-based melanoma identification training intervention.	Online randomized experiment. Pre- and post-test. Fitzpatrick's skin index. Risk behavior diagnostic scale.	A total of 1205 adult participants from the public.	Online melanoma identification game	The game increased melanoma identification accuracy and self-efficacy to perform self-screenings which has encouraging implications for skin cancer prevention behavior, specifically motivating skin self-exam performance.<br><br>Not providing feedback and against expectations feedback appeared to reduce melanoma	Capability Motivation Opportunity

						identification ability.	
	Loerzel et al. (2020) United States of America	To examine the frequency and types of preventive and self-management behaviors reported by participants, as well as report acceptability and usability data for the electronic Symptom Self-Management Training–Chemotherapy-Induced Nausea and Vomiting (CINV) serious game.	Randomized controlled trial. Symptom management checklist developed by investigators.	A total of 80 adults who were aged 60 to 84 years old and newly diagnosed with cancer were recruited.	Online serious game	Serious gaming was shown to be acceptable and useful to older adults.<br><br>The serious game was effective at increasing the number of Chemotherapy-Induced Nausea and Vomiting (CINV) preventive self-management behaviors among older adults with cancer	Capability
	Kato et al. (2008) United States of America, Canada, and Australia	To determine the effectiveness of a video-game intervention for improving adherence and other behavioral outcomes for adolescents	Randomized controlled trial	375 patients who had an initial or relapse diagnosis of a malignancy, currently undergoing	Computer video game	The video-game intervention significantly improved treatment adherence and indicators of	Motivation

		and young adults with malignancies including acute leukemia, lymphoma, and soft-tissue sarcoma.		treatment and expected to continue treatment for at least 4 months from baseline assessment were recruited.		cancer-related self-efficacy and knowledge in adolescents and young adults who were undergoing cancer therapy.	
	Wu et al. (2020) The Republic of China (Taiwan)	To evaluate the effectiveness of using new VR educational aids to improve primary care nurses' treatment decision-making knowledge, details of treatments of oral cancer, and skills in providing pre-treatment educational services to prepare oral cancer patients for their treatments.	Quality improvement pilot project using a pre-post design	17 primary care nurses that care for a large number of oral cancer patients and 44 oral cancer patients were recruited.	VR educational aids with gamification elements	The VR educational aids increased nurses' knowledge about treatment decisions, familiarity with newly developed educational aids, confidence in providing educational services to oral cancer patients. It increased the effectiveness of bedside pre-treatment service, and that they are	Capability Opportunity

						willing to recommend it to their peers.<br><br>VR aids meet cancer patients' needs and provide accurate messages, increase satisfaction and they are willing to use them again.	
	Khalil et al. (2016) United States of America	To evaluate the impact of challenge in a fully automated video game called *Re-Mission* on young adult college students' tendency to perceive the severity of cancer, feel susceptible to cancer, and engage in information seeking behaviour (ISB).	Randomized controlled trial and post-test survey	216 young adults from the public were recruited.	Fully automated video game	The game showed an increase in perceived cancer severity and susceptibility, as well as a seeking of cancer-related information.	Capability Motivation
	Zhu et al. (2021) United States of America	To explore the effectiveness of a narrative-based interactive game about	Randomized controlled trial and post-test survey.	99 adult participants from the public were recruited.	Narrative-based interactive game	The game had a high usability among participants, especially among	Capability Motivation

		the HPV vaccines and cervical cancer as a method to communicate knowledge and perhaps create behavioral outcomes.	The System Usability Survey (SUS) scale.<br>Carolina HPV Immunization Attitudes and Beliefs Scale (CHIAS).			females, and improved the related knowledge.	
	Beale et al. (2007)<br>United States of America, Canada, and Australia	To analyse knowledge gains, participants' ratings of the video game acceptability and credibility, and provide evidence for informed judgments about the specific effects of the game on cancer knowledge.	Randomized controlled trial	375 adolescent and young adult cancer patients.	Online video game	The game group improved more knowledge test scores than the control group.<br><br>Video games can be an effective tool for health education in adolescents and young adults with cancer.	Capability<br>Motivation
	Tong and Hee (2023)<br>Malaysia	To design and evaluate the impact of an educational game on breast cancer awareness	One-group pre- and post-intervention pilot study.	30 Malaysian female university students	Online education game	Using online educational games effectively raised awareness of breast	Motivation

		among female university students in Malaysia.	Online self-administered questionnaire.			cancer among university students.<br><br>Online games can be used as a health educational tool to promote awareness of a topic of interest, as digital games can be accessed easily, game content can be tailored made or updated, and improve participant engagement in learning.	
	Sharma et al. (2018)<br>United States of America	To evaluate the effectiveness of an online, publicly accessible educational intervention that uses a game-based learning (GBL) approach to teach the features of malignant melanomas.	Randomized controlled trial. Quantitative online survey.	106 participants from the public	Online game-based learning intervention	The online game-based learning intervention, is effective at improving malignant melanomas recognition and is preferred over	Motivation

						written education by participants.<br><br>It is an ideal teaching tool to enhance self-screening for malignant melanomas.	
	Beale et al. (2006)<br>United States of America, Canada, and Australia	To explore the self-reported perceptions of young users of a health-oriented videogame about the credibility and acceptability of the videogame used as an adjunct to standard treatment for cancer. To determine effects of the game on self-care and other health-related outcomes.	Randomized controlled trial and follow-up questionnaires	375 patients with cancer, between the ages of 13 and 29.	Video game	More-extensive play with the game results in higher perceived acceptability and credibility.<br><br>The self-care intervention video game would be a useful addition to the psycho-educational resources available to treatment teams.	Motivation

						The median number of completing the missions in the game was low, which might indicate that play is difficult or tiring to maintain, given that many patients are struggling with a serious illness and debilitating side-effects of treatment.	
	Sloan and Saurin (2019) United Kingdom	To design and preliminary evaluation of a browser-based game for high school students and young adults to raise awareness of the nature of cancer.	Pre- and post-test surveys	16 participants from the public	Browser-based game	This game is a useful tool for educating participants about cancer knowledge and raising cancer awareness. Following play, more participants identified the correct answer than the wrong answer.	Capability

	Nirmal et al. (2013) United States of America	To explore the feasibility of developing a culturally tailored video game to increase cervical cancer knowledge among native American, and nonnative Vietnamese and Korean American women with limited English proficiency.	Post-game questionnaire	111 women from the public	Digital health game	Younger women were more likely to play the game, while women who were older and/or who immigrated to US were more likely to need our help in playing the game or simply refused to play.<br><br>Most of the participants stated that they gained some or significant amount of knowledge by playing it.<br><br>The game was more effective in educating them compared to the traditional methods,	Capability

						such as brochures and pamphlets.<br><br>A small percentage of participants had no entertaining experience through playing the game.	
	Ribeiro et al. (2024) Portugal	To field-test a recently developed augmented reality (AR) -based serious game designed to promote skin self-examinations self-efficacy to prevent melanoma.	Quantitative questionnaires. Mobile App Rating Scale (uMARS). Skin Self-Examination Scale.	30 participants from the public	AR-based serious game	The majority of participants considered that the objective quality of the game was high.<br><br>Participants showed statistically significant increases in skin self-examination self-efficacy and intention at follow-up.<br><br>The large majority of participants agreed that the	Capability

						game could have a real impact in melanoma prevention by increasing awareness and knowledge about skin self-examination, and by changing attitudes and motivating users to perform skin self-examination.	
	You et al. (2023) United States of America	To describe game engagement and its associations with learning outcomes, sociodemographics, and health factors in women with advanced cancer receiving a 12-week self-advocacy serious game intervention.	Quantitative surveys. Female Self-Advocacy in Cancer Survivorship (FSACS) Scale. Functional Assessment of Cancer Therapy	38 female patients diagnosed with metastatic breast or advanced gynecologic cancer	Online serious game	Highly engaged participants reported significantly higher 3-month self-advocacy skills of connected strength than relatively lower engaged participants	Capability Motivation

			– General (FACT-G). M.D. Anderson Symptom Inventory (MDASI). Hospital Anxiety and Depression Scale (HADS). Center for Research in Chronic Diseases – Revised survey.			Participants with lower baseline symptom severity were more likely to repeat game scenarios<br><br>Participants' game engagement was overall high.	
	Maganty et al. (2018) United States of America	To evaluate the effectiveness of a game-based learning (GBL) intervention, Tapamole, in improving recognition of the features of melanoma (MM) compared to a written education intervention.	Pre- and post-test surveys	60 participants from the public.	Online game-based learning intervention	The sensitivity for melanoma recognition in the game group was 100% compared to 95% for the pamphlet group. The specificity (40.8% vs 53.3%) and accuracy (60.6% vs 67.2%)	Capability

						of the game and pamphlet groups were similar.<br><br>Game-based learning and written material both improve patient knowledge to a similar extent. However, participants found the game-based intervention more enjoyable than written materials, suggesting people may be more willing to use game-based education materials.	
	Kim et al. (2018) Republic of Korea	To evaluate if patient education using a mobile game may increase drug compliance, decrease physical side effects of	Randomized controlled trial. Questionnaires. Medication Adherence	76 patients with metastatic breast cancer who were planned to	Mobile game	Education using a mobile game led to better patient education, improved drug	Motivation

		chemotherapy, and improve psychological status in breast cancer patients.	Rating Scale (K-MARS). Beck Depression Inventory (BDI). Spielberger State-Trait Anxiety Scale. World Health Organization Quality of Life-BREF Scale.	receive cytotoxic chemotherapy.		compliance, decreased side effects, and better QoL compared with conventional education. There were no significant differences in terms of depression and anxiety scales.<br><br>Mobile games can be used as easy, fun, and effective measures for patient education and have the potential to improve treatment outcomes.	
	Krebs et al. (2019) United States of America	To examine feasibility (recruitment and retention rates), acceptability (patient satisfaction), quitting self-confidence, and other cessation-related indices to guide	Randomized controlled trial	38 cancer patients	Online serious game	Satisfaction with gameplay was largely positive, with most respondents enjoying use, relating to the	Motivation Opportunity

		the development of a cessation app among smokers diagnosed with cancer.				characters, and endorsing that gameplay helped them cope with actual smoking urges.<br><br>Framing of the app as a "game" may have decreased receptivity as participants may have been preoccupied with hospitalization demands and illness concerns.	
Mixed methods (n=4)	Ruiz-López et al. (2019) Norway	To describe a game-based learning app that educates mobile technology users about HPV and cervical cancer and existing preventive methods.	Quantitative descriptive analytics and focus group	40 participants from public who were knowledgeable about cancer were recruited in the quantitative phase.	Game-based mobile app	Participants in the user groups reacted positively toward this educational game.<br><br>Gamification app is beneficial in communicating	Capability

				6 women from public aged between 40-60 were in the focus group.		serious health information, raising awareness, increasing prevention knowledge and self-efficacy, and eliminating misconceptions about HPV and cervical cancer.	
	Wanberg et al. (2023) United States of America	To test the ease of use, usefulness, and satisfaction with the Game-based Learning Avatar-navigated mobile (GLAm) app among young adults.	Qualitative think-aloud play interview and quantitative survey.	23 cervical cancer screening–eligible US residents aged 21 to 29 years.	Game-based Learning Avatar-navigated mobile application	Participants were satisfied with the app format and found it easy to use.<br><br>The app was perceived to be moderately useful to inform and motivate cervical cancer screening, able to ease anxiety about screening through	Capability

						demonstration of the screening process, and the brevity of app components was favored.	
	Kato and Beale (2006) United States of America	To explore whether an action video game about cancer would be acceptable to adolescent and young adult cancer patients as a tool for learning about cancer and self-care during treatment.	Interview and questionnaire. Weinberger Adjustment Inventory (WAI). NEO Five-Factor Inventory (NEO-FFI).	43 adolescent and young adult cancer patients	Video game	Most participants expressed willingness to play the game and a moderate degree of interest in it. Cancer content in the game was not a deterrent for most participants.<br><br>An action video game using cancer themes could be useful to nurses as a tool to improve understanding and self-care of adolescent and	Motivation Opportunity

						young adult cancer patients.	
	Reichlin et al. (2011)<br>United States of America	To determine the acceptability and usability of the alpha version of Time After Time, an interactive decision aid for men diagnosed with localized prostate cancer, in order to inform future iterations of the serious game.	Focus group and quantitative survey.<br>7-point Likert scale.	13 male patients who had already completed treatment for localized prostate cancer	Interactive serious game	The majority of the study participants rated the game as an appropriate decision tool for localized prostate cancer and verified that it meets its goals of increasing focus on side effects and generating questions for the patient's health care team.<br><br>Less positive responses were about the applicability of the game to their own personal cases, reflect their expressed desire for	Capability

						more personalization.	
Co-design method (n=3)	Anderson et al. (2024) United Kingdom	To evaluate the effectiveness of a 'serious game' in increasing awareness of the symptoms of pancreatic cancer and help-seeking intentions within the general public.	Co-design method with quasi-experimental pre-test/post-test evaluation	727 participants from the public were recruited.	Online serious game	The serious digital game can effectively increase public awareness and influence help-seeking intentions.<br><br>The game was also positively evaluated by participants.	Motivation
	Cosma et al. (2015) United Kingdom	To develop and evaluate a digital serious game to raise awareness of prostate cancer among African Caribbean men and to encourage symptomatic men to seek medical attention.	Co-design method with qualitative focus group evaluation. Serious Game Design Assessment (SGDA) Framework	29 African Caribbean men from the public participated.	Online serious game	Participants agreed that the game will have great impact on changing the behaviour of symptomatic men by encouraging them to seek medical attention in a timely manner.<br><br>The game is effective as an intervention for	Capability

						raising awareness, among African Caribbean men, about of prostate cancer risks and symptoms.	
	Cosma et al. (2016) United Kingdom	To introduce and evaluate Prostate Cancer Evaluation and Education (PROCEE), an innovative serious game aimed at providing prostate cancer information and risk evaluation to black African-Caribbean men.	Co-design method with qualitative focus group evaluation.	29 African-Caribbean men from the public	Online serious game	Users defined an easy to use and entertaining game which can effectively raise awareness, inform users about prostate cancer and their risk, and encourage symptomatic men to seek medical attention in a timely manner.<br><br>Users embraced the game and emphasised that it can potentially have a positive impact on	Motivation

						changing user behaviour among high-risk men who are experiencing symptoms and who are reluctant to visit their doctor.	
Design-based research (n=7)	Kayed et al. (2024) Lebanon	To develop an educational radiotherapy training game for medical professionals and students related to lung cancer patients.	Developing a simulation-based serious game using a structured design process.	medical professionals and students	Simulation-based serious game	This serious game provides an educational and empirical space for training and practice that can be used by students, trainees, and professionals to expand their knowledge and skills in the aim of reducing potential errors.	Motivation
	Brown et al. (2014) United Kingdom	To develop an intelligent serious game which will provide users with cues for action for early	Developing an intelligent serious game using a	African and African-Caribbean men during Pre- and Post- Diagnosis	Intelligent serious game	This game will provide knowledge about prostate cancer and encourage African	Motivation

		presentation and diagnosis.	structured design process.	of Prostate Cancer		Caribbean Men to seek medical help.	
	Loerzel et al. (2018) United States of America	To explore how a community advisory board of older adults, their caregivers, and oncology nurses were consulted to develop a serious game for chemotherapy- induced nausea and vomiting (CINV).	Formative evaluation process and semi-structured interview	5 Cancer patients and 3 caregivers, 4 oncology nurses	Serious game	Participants reacted positively to the serious game as an educational tool for patients.	Motivation
	Cerqueira et al. (2025) Portugal	To present and explain the development stages of a mobile app designed to improve health literacy for self-management of oncological diseases. Through the integration of gamification, the app aims to enhance patient engagement and education in an interactive manner.	Design Science in Information Systems and Software Engineering research methodology. Online surveys. System Usability Scale (SUS) Qualitative method	A total of 132 participants, consisting of patients and healthcare professionals	Mobile application with gamification	The usability testing analysis revealed excellent acceptance of PocketOnco, with the gamified elements such as quizzes and reward systems being particularly appreciated for their ability to consistently engage and motivate users.	Motivation

						The application proved to be a viable and attractive solution for both patients and healthcare professionals, suggesting a promising path for future digital interventions in the field of oncology.	
	Magro et al. (2010) United States of America	To develop an interactive e-learning tool and improve cancer screening education among healthcare professionals.	Developmental study and usability testing. Verbal and/or written feedback.	30-60 participants in the healthcare industry	Simulation-based serious game	Simulated education, such as this, can be used on an individual basis at times and locations which are convenient for staff members.<br><br>It also helps the user to retain information, stay engaged with the	Capability Motivation

						task at hand, and learn how to apply the information presented into a real clinical environment.	
	Pereira et al. (2019) Brazil	To develop a serious game that convey educational content about skin lesions to primary care physicians.	Procedural Content Generation (PCG) techniques	Primary care physicians	Online serious game	The game was tested as efficient and satisfactory from the PCG point of view.	Capability
	Swarz et al. (2010) United States of America	To promote strategies and research-tested interventions that physicians and other healthcare workers can implement to overcome barriers and make cancer screening more efficient and cost- effective.	Developmental study and usability testing.	Physicians and other healthcare workers	Web-based learning application in a game format	The iterative development improved the user engagement in the game.<br><br>The game successfully created a more immersive e-learning tool for improving cancer screening training.	Motivation
